# Special Issue on Bioinformatics and Machine Learning for Cancer Biology

**DOI:** 10.3390/biology11030361

**Published:** 2022-02-24

**Authors:** Shibiao Wan, Chunjie Jiang, Shengli Li, Yiping Fan

**Affiliations:** 1 Center for Applied Bioinformatics, St. Jude Children’s Research Hospital, Memphis, TN 38105, USA; yiping.fan@stjude.org; 2 Division of Diabetes, Endocrinology and Metabolism, Department of Medicine, Baylor College of Medicine, Houston, TX 77030, USA; chunjie.jiang917@outlook.com; 3 School of Medicine, Shanghai Jiao Tong University, Shanghai 201620, China; shengli.li@shsmu.edu.cn

Cancer is a leading cause of death worldwide, claiming millions of lives each year. Cancer biology is an essential research field to understand how cancer develops, evolves, and responds to therapy. By taking advantage of a series of “omics” technologies (e.g., genomics, transcriptomics, and epigenomics), computational methods in bioinformatics and machine learning can help scientists and researchers decipher the complexity of cancer heterogeneity, tumorigenesis, and anticancer drug discovery. Particularly, bioinformatics enables the systematic interrogation and analysis of cancer from various perspectives, including genetics, epigenetics, signaling networks, cellular behavior, clinical manifestation, and epidemiology. Moreover, thanks to the influx of next-generation sequencing (NGS) data in the postgenomic era and multiple landmark cancer-focused projects, such as The Cancer Genome Atlas (TCGA) and Clinical Proteomic Tumor Analysis Consortium (CPTAC), machine learning has a uniquely advantageous role in boosting data-driven cancer research and unraveling novel methods for the prognosis, prediction, and treatment of cancer.

This special issue aims to leverage bioinformatics analysis and machine learning to further our understanding of cancer biology in different perspectives. Specifically, Yao et al. [1] identified and validated an Annexin-related prognostic signature and therapeutic targets for bladder cancer. Furthermore, for bladder cancer, Wei et al. [2] demonstrated CPA4 to be a poor prognostic biomarker correlated with immune cells infiltration, and for ovarian cancer, Li et al. [3] identified the RNA modification gene PUS7 as a potential biomarker. Another interesting development is that Serna-Blasco et al. [4] proposed a new measurement called R-score to assess the quality of variants’ calls using liquid biopsies for non-small cell lung cancer. Additionally, for breast cancer, Zainab et al. [5] used a drug–drug interaction network approach to identify estrogen receptor alpha inhibitors, and simultaneously, they revealed the role of persistent organic pollutants in the progression of the breast cancer. Furthermore, Chiu et al. [6] identified a DNA damage repair gene set as a potential biomarker to stratifying patients with high tumor mutational burden. Methodically, Rehman et al. [7] proposed a depth-wise convolutional neural network for architecture distortion-based digital mammograms classification. Obermayer et al. [8] proposed a web-based framework called DRPPM-EASY for integrative analysis of multi-omics cancer datasets.

It is exciting to know that, with the help of integrative bioinformatics analyses, our understanding of multiple cancers such as bladder cancer, ovarian cancer, breast cancer, and non-small cell lung cancer has been remarkably enhanced. Additionally, with the application of machine learning approaches (especially deep learning) and webtool development, our capabilities of extending the analysis and understanding of other less-studied cancers are expected to be consolidated. On the other hand, we are also aware that with heterogeneity and complexity properties, even the internal mechanisms of tumorigenesis for those well-studied cancers [9,10] remain to be further unraveled, let alone the discovery of anticancer drugs and treatment. More integrative, genome-wide, and global-scale studies are required to further shed light on the driving forces behind the tumorigenesis and the development of anticancer drugs and treatments.

In summary, we would like to thank all the authors for the articles published within this special issue who made significant contributions to this special issue, and more importantly, to the better understanding of various cancers as well as cancer-related analysis method improvements. We are eager to see more exciting discoveries in cancer biology with the help of bioinformatics analysis and machine learning in the near future.
